# Patient perspectives on the promptness and quality of care of road traffic incident victims in Peru: a cross-sectional, active surveillance study

**DOI:** 10.12688/f1000research.2-167.v1

**Published:** 2013-08-09

**Authors:** J Jaime Miranda, Edmundo Rosales-Mayor, D Alex Quistberg, Ada Paca-Palao, Camila Gianella, Pablo Perel, Luis Lopez, Diego Luna, Pablo Best, Luis Huicho, PIAT Working Group

**Affiliations:** 1 Programa de Investigación en Accidentes de Tránsito, Salud Sin Límites Perú, Lima, Peru; 2 School of Medicine, Universisdad Peruana Cayetano Heredia, Lima, Peru; 3 CRONICAS, Center of Excellence in Chronic Diseases, Universidad Peruana Cayetano Heredia, Lima, Peru; 4 EDHUCASALUD, Asociación Civil para la Educación en Derechos Humanos con Aplicación en Salud, Lima, Peru; 5 Centro de Trastornos Respiratorios del Sueño (CENTRES), Clínica Anglo Americana, Lima, Peru; 6 Grupo de Investigación en Sueño (GIS), Lima, Peru; 7 Hospital Clínic de Barcelona, Barcelona, 08036, Spain; 8 Department of Epidemiology, School of Public Health, University of Washington, Seattle WA, 98195-7236, USA; 9 Harborview Injury Prevention & Research Center (HIPRC), University of Washington, Seattle WA, 98104-2499, USA; 10 Faculty of Epidemiology and Population Health, London School of Hygiene & Tropical Medicine, London, WC1E 7HT, UK; 11 Dirección de Formación Profesional y los Recursos Humanos, Ministerio del Trabajo y Promoción del Empleo, Lima, Peru; 12 Departamento de Ciencias Sociales y Políticas, Universidad del Pacífico, Lima, Peru; 13 Asociación Civil, Gobierno Coherente, Lima, Peru; 14 School of Public Health and Administration, Universidad Peruana Cayetano Heredia, Lima, Peru; 15 Department of Pediatrics, Instituto Nacional de Salud del Niño, Lima, Peru; 16 School of Medicine, Universidad Nacional Mayor de San Marcos, Lima, Peru

## Abstract

**Background: **Road injuries are the second-leading cause of disease and injury in the Andean region of South America. Adequate management of road traffic crash victims is important to prevent and reduce deaths and serious long-term injuries.

**Objective: **To evaluate the promptness of health care services provided to those injured in road traffic incidents (RTIs) and the satisfaction with those services during the pre-hospital and hospital periods.

**Methods: **We conducted a cross-sectional study with active surveillance to recruit participants in emergency departments at eight health care facilities in three Peruvian cities: a large metropolitan city (Lima) and two provincial cities (an urban center in the southern Andes and an urban center in the rainforest region), between August and September 2009. The main outcomes of interest were promptness of care, measured by time between injury and each service offered, as well as patient satisfaction measured by the Service Quality (SERVQUAL) survey. We explored the association between outcomes and city, type of health care facility (HCF), and type of provider.

**Results**: We recruited 644 adults seeking care for RTIs. This active surveillance strategy yielded 34% more events than anticipated, suggesting under-reporting in traditional registries. Median response time between a RTI and any care at a HCF was 33 minutes overall and only 62% of participants received professional care during the initial “golden” hour after the RTI. After adjustment for various factors, there was strong evidence of higher global dissatisfaction levels among those receiving care at public HCFs compared to private ones (odds ratio (OR) 5.05, 95% confidence interval (CI) 1.88-13.54). This difference was not observed when provincial sites were compared to Lima (OR 1.41, 95% CI 0.42-4.70).

**Conclusions:** Response time to RTIs was adequate overall, though a large proportion of RTI victims could have received more prompt care. Overall, dissatisfaction was high, mainly at public institutions indicating much need for improvements in service provision.

## Introduction

### Background

Worldwide, road traffic incidents (RTIs) constitute the primary cause of death due to injuries, the tenth leading cause of all deaths, and the ninth leading contributor to the global burden of disease ^1^. Latin America and Peru are not exceptions to these statistics ^2, 3^. Adequate management after the occurrence of a traffic incident can decrease the probability of death and disability, limit the severity caused by injury and ensure that survivors are optimally reintegrated into the community ^4^. In high-income countries, studies have shown that of those that die due to a RTI, 50% will die within minutes after the incident or on the way to the hospital, 15% die within the first 4 hours, and 35% die after 4 hours ^5^. In low- and middle-income countries (LMIC), the majority of deaths occur among victims before arriving at a health care facility (HCF), indicating that many deaths and complications could be prevented via adequate initial management and response ^5– 8^. The experiences of developing countries with regards to evaluating the care of RTI victims are scarce and unsystematic ^9^. Providing quality health care services to RTI victims is also important, ensuring that they are at the center of attention of all services offered ^10^. Patients and users have a wide spectrum of cultures and attitudes, and both demand and hope for quality in all scientific, technical and humanistic aspects ^11, 12^.

### Importance

Determining the response and quality of emergency services in LMICs is important for understanding how to strengthen these services in low-resource areas. One of the first steps to approach the problem of RTIs is to rely on solid information at a national level ^13^, and RTIs have been considered a research priority for Peru ^14^.

### Goals

To evaluate how well the promptness of care complies with the recommendations for emergency care services during the pre-hospital and hospital periods as suggested by the US Department of Transportation (USDOT) and the National Highway Transportation Safety Administration (NHTSA) in the USA ^15^, and how much satisfaction the victims have with respect to the care received. Additionally, we sought to assess the association between the city and type of HCF where the victim was cared for with the quality of attention received.

## Methods

### Study design and setting

We conducted a cross-sectional study over a period of four weeks, August-September 2009, in the Emergency Departments of 8 HCFs in Ayacucho (South Andes), Lima (Peru’s capital) and Pucallpa (rainforest region). These regions include urban areas with higher socioeconomic status and complex referral hospitals (Lima), and inner cities with a lower level of socioeconomic development (Ayacucho and Pucallpa) ^16^. Both public (hospitals) and private (clinics), were selected on the basis of their closeness to each city’s arterial routes (highways and expressways) and the volume of RTI victims they received. The average number of potential eligible subjects receiving care at the selected facilities, for 2007–2008, ranged from 43 to 150 RTI victims per month. Based on these figures, we assumed that approximately 60 patients per site per month would be expected between August and September 2009.

### Participants

The study population was adult victims of RTIs seeking health care for injuries at the selected HCFs, arriving on their own or carried by others (e.g. physician, firefighter, ambulance service, police, or other road user). We did not recruit anyone that presented in an altered state of consciousness or speech, since they were not in a condition to respond to the interview; therefore we excluded critically ill patients.

The protocol was reviewed and approved by the ethical committee of Peru’s Instituto Nacional de Salud and by ethics committees at the HCF if they had one. Written consent was obtained from each participant.

### Data collection and processing

Active surveillance was used to identify all possible participants by having research interviewers present 7 AM to 1 PM and 1 PM to 7 PM, Monday to Saturday. Subjects were interviewed without interrupting their care by staff. We visited patients at home if they had been admitted or discharged during the hours or days not covered by interviewers.

We assessed whether pre-hospital care was provided by untrained personnel (other road users, police, or municipal patrol guards also called “ *Serenazgo*”) or by trained ones (firefighters and/or health professionals, assuming that firefighters receive first aid training). The promptness of attention was evaluated through an interviewer–administered, semi-structured questionnaire about waiting times before receiving care during the pre- and hospital periods.

The survey questionnaire was pilot-tested beforehand. There was sufficient evidence that there was adequate comprehension of the SERVQUAL instrument and its face validity. The modified Service Quality questionnaire (SERVQUAL) ^17, 18^ had a Cronbach alpha of 0.96, indicating good internal consistency and reliability of the instrument in the target population.

### Outcome measures

We defined the pre-hospital period as starting from the moment the incident occurred until the moment prior to arrival at the HCF, and hospital period as starting from arrival at the HCF onwards. Waiting times were self-reported by subjects. The quality of care during the pre-hospital period was evaluated on a 5-point Likert-type scale ranging from “Very Poor Care” to “Very Good Care”. To evaluate the pre-hospital quality of care for the purpose of analysis, we combined the categories of “Very poor care” and "Poor care".

The Service Quality (SERVQUAL) questionnaire was used to determine customer satisfaction ^17, 18^ with the HCF service received. SERVQUAL proposes a “discrepancy model” where the difference between expectations of what should be received versus perceptions of what was actually received at the point-of-care indicates the quality of service ^18– 20^. We used a modified SERVQUAL using 18 (out of 22) pairs of questions that have the most relevance to health care ^19^, and that has been frequently used to evaluate quality of health care in Peru ^21– 25^. SERVQUAL evaluates satisfaction in five dimensions: i) tangibles: appearance of the physical facilities, equipment, staff, and communication materials; ii) reliability: delivering the service exactly as promised or as agreed; iii) responsiveness: willingness to quickly and immediately assist customers at a given opportunity; iv) assurance: courtesy and the ability to convey credibility without risks or prejudice when providing service; and v) empathy: willingness to put oneself in the place of another, to think first of the patient, and to serve according to specific characteristics and situations.

In addition to SERVQUAL, we examined other variables that demonstrate evidence of deficiencies in distinct processes of patient care, including the explanation of the diagnosis and the treatment of the victim using a structured questionnaire (see Data File). Finally, we asked the participants which primary aspect of the HCF where they attended should be improved.

### Data analysis

The hospital user satisfaction score for each of the five SERVQUAL dimensions was determined by taking the difference between expectations and perceptions (very satisfied <0, moderately satisfied=0, somewhat or moderately dissatisfied >0 and ≤2, and extremely dissatisfied >2). The global score was calculated by taking the average scores of the 5 dimensions described above. Then, for analysis, satisfaction scores were dichotomized as satisfied (very satisfied and satisfied) and dissatisfied (somewhat or moderately dissatisfied and extremely dissatisfied).

We conducted a descriptive analysis of the frequencies and distribution of the variables and tested for statistically significant differences between groups using the Kruskal-Wallis test for outcomes with medians and *χ ^2^* tests for outcomes with frequencies. We used a multivariable logistic regression to evaluate the association between the degree of dissatisfaction with city type and facility type. For this analysis, we first developed models adjusted for age and sex (Model 1) and then adjusted for potential confounding variables defined *a priori*, such as education and employment (Model 2). Model 2 was also adjusted for the type of facility and type of city depending on the outcome of interest. We report odds ratios (OR) and 95% confidence intervals (95% CI). Statistical analysis was conducted with Stata 10 (STATA Corp, College Station, TX, USA).

## Results

### Characteristics of study subjects

**Figure 1.**
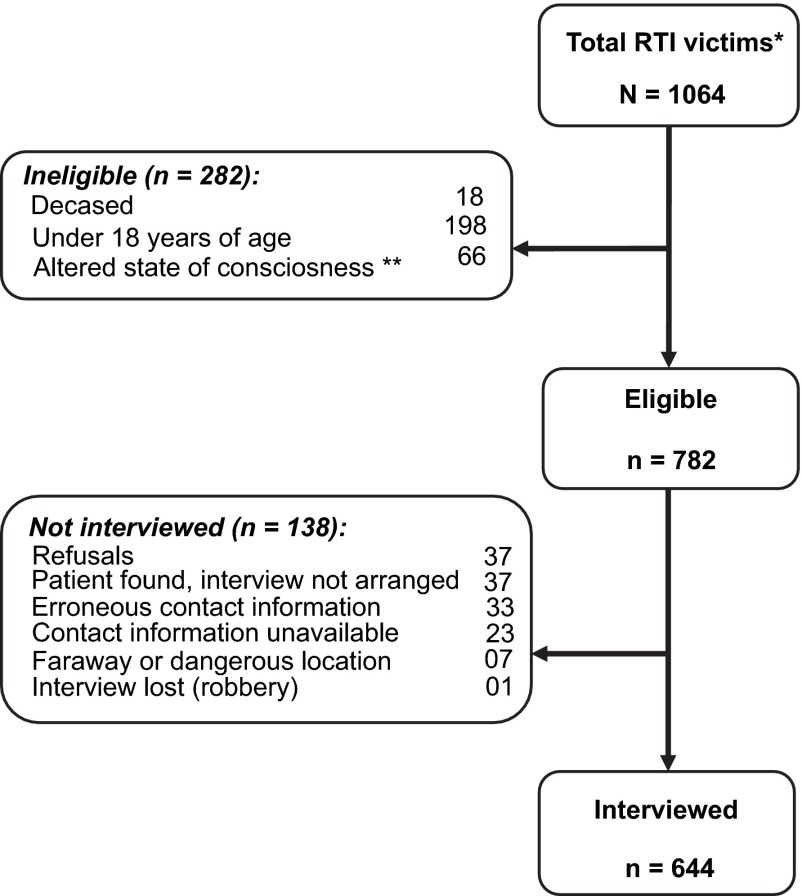
Flowchart of study participation. * Victims admitted to emergency services of health care facilities (HCFs) included in the study. This includes all patients cared for in the emergency department and discharged with or without previous hospitalization. ** Altered states of consciousness and speech directly attributable to the road traffic incident (RTI; for example, encephalocranial traumas) or not (for example, alcohol intoxication, Alzheimer’s, dementia, senility, etc.) that affected the perception of the event and did not allow the respondent to answer the questionnaires.

Six public HCFs, one in Ayacucho, two in Pucallpa and three in Lima; and two private HCFs, both in Lima, participated in the study. A total of 1064 RTI victims sought care in the eight emergency departments during the study period ( Figure 1). Of them, 644 participated in the study: response rate 82%, 46% female, mean participant age 37.3 (SD 15.7 years), age range 18–88 years.

Of those eligible to participate but were not interviewed, 40% were female, mean age 39.4 (SD 15.7 years), age range 18–87 years. There were no significant differences between participants and non-participants in terms of gender or age. There were no significant differences between participants by cities with respect to education or employment.

### Promptness to care during the pre-hospital period

***Profile of first responders after the RTI event*.** According to the victims, another road user was the first responder in 72% of the cases ( Table 1). The median time to first response was 2 minutes (inter quartile range (IQR) 1–5) in all three cities, with no evidence of a difference in time (p=0.089). Other road users had the shortest median response time ( Table 2) at 2 minutes (IQR 1–3) and firefighters (in Lima only) had the longest median time at 15 minutes (IQR 10–20).

**Table 1.** Descriptive statistics of participants.

	Lima	Pucallpa	Ayacucho	Total
	N=444	N=142	N=58	N=644
Male	54	61	39	54
Age (Mean, SD)	38.8 (16.3)	33.3 (13.7)	36.2 (13.4)	37.3 (15.7)
Treated in public HCF	73	100	100	81
**Education level**				
Any post-secondary	33	32	27	33
Completed secondary	39	37	25	37
Incomplete secondary	12	17	7	13
Completed primary	8	10	14	9
Incomplete primary	6	4	15	6
None	2	0	12	2
**Employment status**				
Unemployed	4	6	5	5
Temporary	43	56	17	43
Permanent	37	12	32	31
Homemaker	14	18	39	17
Student	2	8	7	4
**First pre-hospital care provider**				
None	8	11	24	10
Other road user	68	84	71	72
Police or *Serenazgo*	17	5	5	13
Ambulance	2	0	0	1
Firefighters	5	0	0	3
**First hospital care provider**				
Physician	71	7	32	53
Nurse	23	30	29	25
Medical student	1	2	8	2
Nurse technician	5	61	31	20

All numbers are proportions (%) of total N for each column except where noted. *Serenazgo*: municipal patrol guards.

**Table 2.** Median time (minutes) and interquartile range (IQR) to obtain initial care by provider type in each city within the pre-hospital and hospital periods.

	Lima	Pucallpa	Ayacucho	P-value ^*^	Total
*Pre-hospital period*	N=374	N=117	N=38		
Firefighters	15 (10–20)	^†^	^†^	^‡^	15 (10–20)
Other ambulance service	5 (3–30)	^†^	^†^	^‡^	5 (3–30)
Police & *serenazgo* ^**^	3 (2–10)	5 (5–10)	47 (3–90)	0.1816	5 (2–10)
Other road users	1 (1–3)	2 (1–5)	2 (1–7,5)	0.0001	2 (1–3)
***Hospital period***	**N=401**	**N=131**	**N=53**		
Physician	5 (2–15)	4 (2–10)	2 (1–5)	0.0033	5 (2–15)
Nurse	5 (2–10)	5 (2–5)	10 (4–23)	0.0573	5 (2–10)
Medical student	1 (1–1)	10 (9–30)	10 (5–15)	0.0096	5 (1–10)
Nurse technician	2 (1–5)	5 (2–9)	5 (2–5)	0.1669	5 (2–9)

^*^ P-value calculated with Kruskal-Wallis test.

^**^ *Serenazgo*: municipal patrol guards.

^†^ No subjects were served by these providers.

^‡^ Not applicable.

N.B. The number is higher in the hospital period than the pre-hospital period as some participants could not remember details about the pre-hospital period.

**Figure 2.**
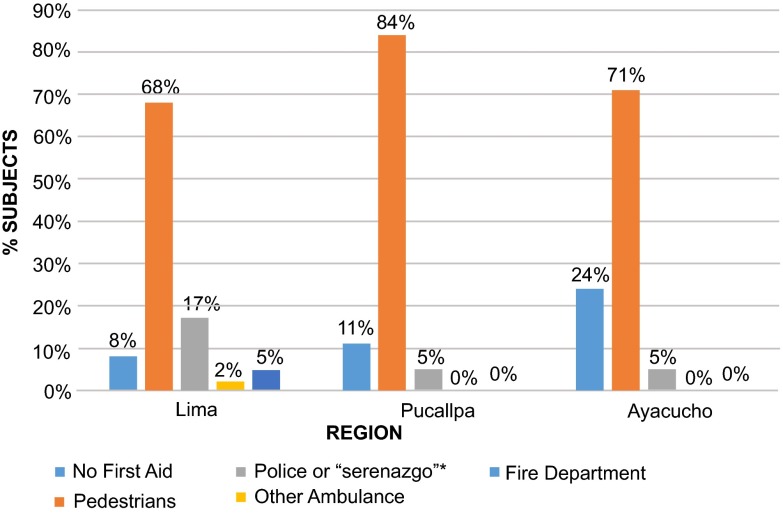
Profile of individuals that provided initial care during the pre-hospital period, by city. There were a total of 644 patients. * *Serenazgo*: municipal patrol guards.

***First response by trained personnel*.** With regards to trained personnel as first care providers, firefighters and ambulance services represented 4% of first responders in Lima ( Figure 2) with a median response time of 10 minutes (IQR 5–20). There were no instances of trained personnel being the first responders in the other locations.

***Care received after first response*.** Only 21% of the respondents who were not treated by a trained first responder received further care from a trained provider before arriving at the HCF, though 2% did not respond to the question. The median additional time-to-trained-care by a trained provider, following a first response by any non-trained care provider, was 15 minutes (IQR 10–20). There was strong evidence of a difference in time-to-trained-care (p=0.0002) between cities with Lima at 15 minutes (IQR 10–20), Pucallpa at 10 minutes (IQR 6–10), and Ayacucho at 120 minutes (IQR 20–240).

***Global care during the pre-hospital period*.** Overall, 22% of the victims reported having received some sort of care by trained responders, be it first response or afterwards (27% in Lima, 7% in Pucallpa and 18% in Ayacucho). Taking together the entire pre-hospital period, 66% never received care by a qualified responder before arriving to a HCF (75% in Lima, 92% in Pucallpa and 74% in Ayacucho, p<0.001).

***Observed versus recommended standard pre-hospital response times*.** Taking into account the response time standards of the DOT and NHTSA for pre-hospital care, only 35% of RTI victims received care during the first 8 minutes or less. Only 10% of the victims arrived at the HCF within the response time standard of 38 minutes after the incident. Cities differed in meeting this standard: 13% in Lima, 4% in Pucallpa and 5% in Ayacucho; p<0.002. The median time from the occurrence of the RTI until arrival at the HCF was 30 minutes (IQR 15–45). When examining this time by city we found strong evidence of a difference between cities (p=0.0001); the median time in Lima was 30 minutes (IQR 20–45), 20 minutes (IQR 10–35) in Pucallpa, and 40 minutes in Ayacucho (IQR 20–270).

### Promptness to care received during the hospital period

**Figure 3.**
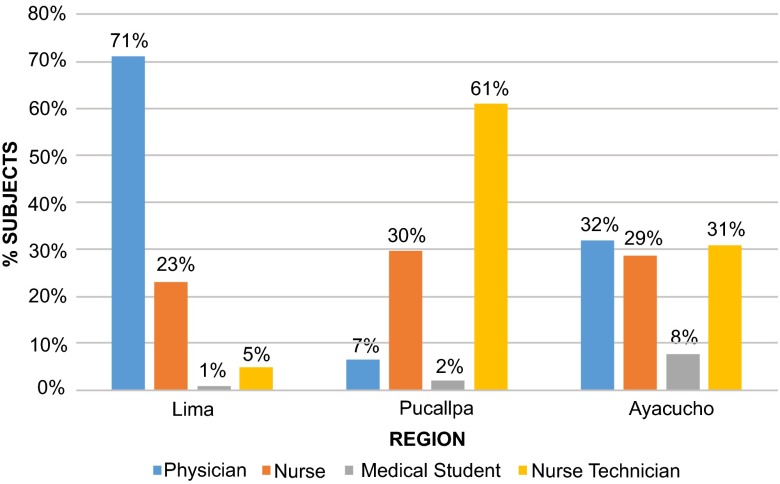
Caregiver that provided initial care during the intra-hospital period after the incident. There were a total of 644 patients.

***Provision of care at HCF*.** At arrival at the HCF, the type of professionals involved in the provision of care varied dramatically between cities ( Figure 3). For all victims, the median time of response-to-initial-care after arriving at a facility was 5 minutes (IQR 2–10). There was no evidence of difference in this time by city (p=0.1547) or by type of HCF (p=0.3327). When disaggregated by professions, the time of response-to-initial-care was different for doctors and medical students but not for nurses and nurse technicians ( Table 2).

When a physician was not the initial caregiver, the additional median waiting time to see a physician was 10 minutes (IQR 5–20). Reported times for Lima were 7 minutes (IQR 3–20), 10 minutes (IQR 5–20) in Pucallpa and 10 minutes (IQR 10–20) in Ayacucho, though these different times were not borderline statistically significant (p=0.0505).

***Total elapsed time from the road traffic incident until intra-hospital care*.** The overall median time from the RTI until any care was received at a HCF was 33 minutes (IQR 22–65). In Lima the overall median time was 35 minutes (IQR 23–70), 25 minutes (IQR 17–42) in Pucallpa, and 52.5 minutes (IQR 27–275) in Ayacucho. Care at a HCF was received by 62% of the participants during the first “golden hour”. The proportion in Lima that received care in these first 60 minutes i.e. within the “golden hour” was 60%, 77% in Pucallpa, and 44% in Ayacucho (p<0.001).

### Patient satisfaction

***Satisfaction with care received during the pre-hospital period*.** Less than 10% of the victims reported that their first responder provided them with a poor care service. By type of first responder, 6% of the victims reported poor care by pedestrians, 7% by firefighters, 8% by the police, 8% by other ambulance services, and 9% by *serenazgo*.

***Satisfaction with care received during the intra-hospital period*.** The response rate to this survey component was 99%. In all cities and public HCFs we found a high proportion, around 75% or more, of victims that were dissatisfied. This was observed in each of the specific domains as well as in the global aggregate ( Table 3). The highest proportions of satisfaction were at private HCFs, but only in the domains of tangibles, assurance, and empathy ( Table 3).

**Table 3.** Proportion of dissatisfaction, score for each of the domains, both individual and global scores, listed in the modified Service Quality questionnaire by city and health care facility type.

	Lima n=442 %	Pucallpa n=139 %	Ayacucho n=59 %	P-value ^*^	Public n=518 %	Private n=122 %	P-value ^*^
Global	89	96	100	0.002	95	78	<0.001
Tangibles	73	88	95	<0.001	84	53	<0.001
Reliability	84	85	98	0.013	89	70	<0.001
Responsiveness	83	76	90	0.048	86	67	<0.001
Assurance	77	77	92	0.035	83	59	<0.001
Empathy	76	78	92	92	83	56	<0.001

^*^ P-value calculated with χ ^2^ for trend.

In an adjusted multivariable analysis ( Table 4) there were no evidence of an association between city and satisfaction, except in the domain of responsiveness. Compared to Lima, provincial HCFs showed more satisfaction in responsiveness (OR 0.32, 95% CI 0.15–0.68). Initially, the chances of lower satisfaction, in both global and specific domains, was 3 to 4-fold higher in the provinces compared to Lima, but these estimations became attenuated in the fully adjusted model ( Table 4).

**Table 4.** Association of dissatisfaction, global and by domain, with city.

Lima (reference) vs. Provinces ^*^	Model 1 ^**^		Model 2 ^¶^	
	OR	(95% CI)	OR	(95% CI)
Global	3.90	(1.64–9.32)	1.41	(0.42–4.70)
Tangibles	3.46	(2.06–5.83)	1.57	(0.72–3.39)
Reliability	1.52	(0.91–2.54)	0.89	(0.39–2.08)
Responsiveness	0.79	(0.51–1.22)	0.32	(0.15–0.68)
Assurance	1.29	(0.84–1.97)	0.63	(0.32–1.25)
Empathy	1.43	(0.93–2.21)	0.55	(0.27–1.12)

^*^ Provinces: Ayacucho y Pucallpa.

^**^ Model 1 adjusted for age and sex.

^¶^ Model 2 adjusted for age, sex, education, employment, type of establishment, caregiver that provided care in the facility, time from the event to facility, and explanation of diagnosis to the patient.

There was strong evidence of an association between dissatisfaction and the type of HCF, which was observed in all domains of care provision. Compared to private services, participants who received care at public HCFs were 4 to 7 times more likely to report dissatisfaction. These estimates became stronger in fully adjusted models, and the magnitude of the odds estimates nearly doubled in the domains of responsiveness and empathy ( Table 5).

**Table 5.** Association of dissatisfaction, global and by domain, with type of health care facility.

Private (reference) vs. Public HCF	Model 1 ^*^		Model 2 ^¶^	
	OR	(95% CI)	OR	(95% CI)
Global	5.01	(2.82–8.90)	5.05	(1.88–13.54)
Tangibles	4.82	(3.14–7.41)	4.97	(2.41–10.23)
Reliability	3.39	(2.10–5.47)	3.93	(1.75–8.84)
Responsiveness	2.99	(1.90–4.70)	5.12	(2.34–11.22)
Assurance	3.38	(2.20–5.20)	3.81	(1.87–7.77)
Empathy	3.92	(2.55–6.01)	6.56	(3.17–13.61)

^*^ Model 1 adjusted for age and sex.

^¶^ Model 2 adjusted for age, sex, education, employment, city, caregiver that provided care in the facility, time from the event to facility, and explanation of diagnosis to the patient.

### Explanation of diagnosis and treatment of the patient

Nearly 20% of participants reported that their diagnosis was not explained to them: 12% in Lima, 37% in Pucallpa, and 26% in Ayacucho (p<0.001). The person explaining the diagnosis was usually a physician (95%). For the rest of the RTI victims, it was a nurse (3%) or medical student (2%). A lower proportion of subjects (16%) reported a lack of explanation about the treatment or procedures indicated. In Lima, 12% reported their treatment was not explained, 24% in Pucallpa, and 31% in Ayacucho (p<0.001).

### Areas for improvement according to patient’s perception

The response rate to this question was 90%. The majority, 58% of RTI victims, reported that promptness of care was the top ranking HCF’s aspect that should improve. This was followed by conduct or behavior towards patients, and then by improvements in equipment and tools ( Table 6). Most of the aspects highlighted for improvement were similar between public and private HFCs. While only a small difference (6% vs. 0%) the provision of more administrative and medical information ranked worse in private facilities compared to public ones.

**Table 6.** Aspects that should be improved in health care facilities (HCFs) according to patients, by city and type of establishment.

	City			Type of HCF		
	Lima n=390 %	Pucallpa n=138 %	Ayacucho n=59 %	Private n=85 %	Public n=502 %	Total n=587 %
Promptness of care	63.1	46.4	55.9	57.6	58.6	58.4
Conduct towards patients	10.8	24.6	23.7	15.3	15.3	15.3
Equipment & tools	7.7	23.2	1.7	3.5	11.9	10.7
Layout of facilities	9.5	0.7	0	5.9	6.6	6.5
Knowledge of health care personnel	4.3	5.1	17.0	4.6	6.0	5.9
Provision of more administrative and medical information	1.5	0	0	5.9	0.2	1.0
More personnel	1.0	0	0	1.2	0.6	0.7
Lack of medications at HCF’s pharmacy	0.5	0	1.7	0	0.6	0.5
Less administrative processes	0.5	0	0	2.4	0	0.3
Cleanliness of premises or equipment	0.5	0	0	1.2	0.2	0.3
Decreasing cost of care	0.3	0	0	1.2	0	0.2
Coordination or orderliness among personnel for provision of care	0.3	0	0	1.2	0	0.2

Emergency response times, response provider and patient satisfaction data for individuals in three Peruvian health care facilities. File 1: Service quality questionnaire and promptness of health care formulary (Spanish) used to collect data from patients involved in road traffic incidents in three different Peruvian cities between August – September 2009. 
File 2: Service quality questionnaire and promptness of health care data collected from patients involved in road traffic incidents in three different Peruvian cities between August – September 2009. City: 0 ‘Lima’ 1 ‘Pucallpa’ 2 ‘Ayacucho’; type of health care facility (HCF): 0 'Private' 1 'Public'.

## Discussion

By taking advantage of active surveillance at HCFs that had a high volume of RTIs in three Peruvian cities, this study demonstrated notable deficiencies in the promptness and quality of care in the management of RTI victims. These include untrained first responders, a lengthy elapsed time until receiving attention by qualified caregivers, and above all, a marked dissatisfaction with the care received. Strong evidence of dissatisfaction was observed in public HCFs as compared with private institutions but not by geographical location, with the exception of responsiveness. Participants from Lima, the largest city in the country, were more likely to show dissatisfaction in terms of responsiveness as compared with other settings.

The implications of these findings are important for improving emergency services in these locations. Considering that other road users were usually the first responders and that the victims often had to wait until they arrived at a HCF to receive their first care from trained personnel, it is likely that most victims are not receiving adequate care in the pre-hospital period and so not meeting standards suggested by the USDOT and the NHTSA in the USA ^15^. Ideally, the response time should be 8 minutes or less in at least 90% of all care. In our study, the median time from an RTI event to any form of care received at a HCF was 33 minutes. Indeed, 66% never received care by a qualified responder before arriving to a HCF. A previous study by the Assisted Emergency Transport System of ESSALUD in Lima, Peru by Lira *et al.* ^26^ looked at the times from ambulance departure to arrival to an RTI event. They found that only 7/258 events (2.7%) met the target response time (defined as within 8 minutes in at least 90% of events).

The differences between the aforementioned international standards and those we observed could be due to incompletely developed service networks and assistance models in our study setting. In Peru these are comprised of a fragmented rendering of services and a disjointed reference and counter-reference system ^27^. It is important to note that in Peru pre-hospital care is limited to the care and transport of patients to a HCF, thus the adequate conditions of opportunity (i.e. early treatment, ideally as close to the RTI as possible), quality, and appropriateness (i.e. the best possible care for a given scenario) are limited and not necessarily guaranteed.

Though the reasons for the delayed responses were not an objective of this study, the findings were distinct enough in each context, i.e. city or type of HCF, that more research should be dedicated to understanding response time delays in the different locations and facility types. In relation to the notable delay observed in Ayacucho, one might speculate that the type of incidents and victims differ substantially than those in other cities. For example, these incidents could have occurred outside the city and/or could be related to highway incidents, which is suggested by the statistics from the police (Policía Nacional del Perú) ^28^ and another study by our Program ^3^. The factors associated with a variation in the quality of care of patients that suffered from a RTI were diverse and also intimately related to the problems faced by health care systems. These factors are variable and depend on the context of where care is offered-from any immediate care given after an incident, continuing through the specialized care during the hospital period, and also the rehabilitation of the victims.

The SERVQUAL instrument used in this study sheds light on remarkable statistics of global dissatisfaction, close to 90% in all cities and all types of HCFs. Thus, these findings increase our understanding of the most salient aspects of dissatisfaction with trauma care in Peru. One method to improve the quality of hospital care that has demonstrated cost-effectiveness is the implementation of improvement programs for trauma and emergency services laid out in the Guidelines for Essential Trauma Care, published by the WHO ^4^. Their application to our health system has been insufficient at present ^29^.

### Strengths and limitations

One of the strengths of the study was the active surveillance strategy we enacted. First, the coverage of the study (n=644) exceeded the volume of victims expected; as described in the methods approximately 60 patients per site per month were expected, 480 in all sites. Our active surveillance strategy yielded 34% more events than anticipated and unveils ongoing underreporting in traditional registries ^30^. Second, the response rate obtained (82%) indicates that the methodology used was acceptable for fulfilling the objectives of the study. This strategy, therefore, constitutes a high-quality tool that could be replicated to evaluate RTIs, for the measurement of quality in other areas and as a tool linked to a continual evaluation process with the objective of improving the quality of services. Additionally, being able to carry out an active monitoring of victims of RTI that sought care at HCFs enabled us to obtain information that truly occurred to the said victims during the period of interest.

Some limitations deserve consideration. The HCFs included in the study were restricted to larger institutions, specifically those with a high volume of RTI victims. Yet, knowledge generated by this study might inform other areas of Peru and other countries in the region with regards to gaps in management and quality of care in trauma services. Another limitation of this study is that all recorded times were self-reported by the injured or his/her family with a tendency to round the numbers. This could vary according to the seriousness of the injuries of the victim, and one could argue that there are alternative mechanisms of measurement of time that might avoid the potential biases of self-reporting. For example, a better way to estimate times in promptness of care is to use the exact date and time that an emergency call was registered and the moment that the ambulance leaves the scene. Given, however, that the majority, 66%, of those affected did not receive care from a qualified caregiver until arriving at a HCF, self-reporting was the most valid strategy to obtain the information of interest.

## Conclusion

In conclusion, our study revealed important limitations of the health system that need to be strengthened if the objective of limiting death and disability of RTI victims is to be fulfilled. This was demonstrated by the low quality of care provided in terms of satisfaction with hospital care, especially outside Lima and in public facilities. Because of this, improving the capacity of care of RTI victims in the pre-hospital period with knowledge of first aid, particularly by other road users; reducing the wait time for care, particularly by trained caregivers; and improving the quality of hospital care are indispensable.
