# Supplementary material for: Patient perspectives on the promptness and quality of care of road traffic incident victims in Peru: a cross-sectional, active surveillance study
Source: F1000Res. 2013 Aug 9;2:167. [Version 1] doi: 10.12688/f1000research.2-167.v1 (PMC3814912; doi:10.12688/f1000research.2-167.v1)
Supplement: Emergency response times, response provider and patient satisfaction data for individuals in three Peruvian health care facilities. — File 1: Service quality questionnaire and promptness of health care formulary (Spanish) used to collect data from patients involved in road traffic incidents in three different Peruvian cities between August – September 2009. File 2: Service quality questionnaire and promptness of health care data collected from patients involved in road traffic incidents in three different Peruvian cities between August – September 2009. City: 0 ‘Lima’ 1 ‘Pucallpa’ 2 ‘Ayacucho’; type of health care facility (HCF): 0 'Private' 1 'Public'. [file f1000research-2-902-s0000.tgz › PIAT_Quality_of_Care_Peru___survey.pdf]

Salud Sin Límites Perú

NÚMERO DE ENCUESTA ☐☐☐☐

**ANEXO 10: <u>Entrevista sobre atención pre e intra-hospitalaria de accidentes de tránsito. Paciente.</u>**

*Las instrucciones para el entrevistador se encuentran en itálicas*.

Buenos días/tardes, señor/señora/señorita/joven/jovencita, nos encontramos realizando un estudio sobre accidentes de tránsito. Para ello le agradeceré nos conceda unos minutos de su tiempo para contestar una encuesta. Muchas gracias.

| | | | |
|---|---|---|---|
| **Fecha de entrevista:** | ☐☐ / ☐☐ / ☐☐ *(dd/mm/aa)* | | |
| **Hora de inicio:** | ☐☐ : ☐☐ (de 00:01 a 24:00 hrs) | **Hora final:** | ☐☐ : ☐☐ (de 00:01 a 24:00 hrs) |
| **Rechazo a contestar:** | 0. No ( ) 1. Si ( ) | | |

- ***<u>Los pacientes serán ubicados en:</u>*** *Casas de personas que hayan sufrido accidentes de tránsito y hayan sido atendidos en Lima, Ayacucho y Huancayo. Personas encontradas en la base de datos de la asociación de accidentados, por la información de SOAT o información de la historia clínica revisada en los establecimientos de salud. Las preguntas dirigidas sobre el establecimiento de salud serán relacionadas al lugar en donde lo atendieron en un primer momento luego de ocurrido el accidente.*

**<u>Preguntas control:</u>**

| | | | |
|---|---|---|---|
| **Nombre paciente:** | ………………………………………………………………………………….. | | |
| **Fecha de nacimiento:** | ☐☐ / ☐☐ / ☐☐ *(dd/mm/aa)* | **Edad:** | ☐☐☐ (años cumplidos) |
| **Sexo:** | 1. Hombre ( ) 2. Mujer ( ) | | |
| **Grado de instrucción:** | 0. Primaria completa ( ) 1. Secundaria completa ( )<br>2. Instituto Superior ( ) 3. Universidad ( )<br>4. Sin primaria completa ( ) 5. Sin secundaria completa ( )<br>6. NS/NR ( ) | | |
| **Empleo:** | 0. Desempleado ( ) 1. Ama de Casa ( )<br>2. Temporal ( ) 3. Permanente ( )<br>4. NS/NR ( ) | | |
| **Región:** | 0. Lima ( ) 1. Ayacucho ( ) 2. Junín ( ) | | |
| **Distrito del domicilio:** | ……………………………………………… | | |
| **Zona de domicilio:** | 0. Rural ( ) 1. Urbano – ciudad ( ) | | |
| **Hospital, centro de salud o puesto de salud en el que fue atendido luego del accidente:** | ………………………………………………………………………………………………… | | |
| **Fecha del accidente:** | ☐☐ / ☐☐ / ☐☐ *(dd/mm/aa)* | | |
| **Hora del accidente:** | ☐☐ : ☐☐ (de 00:01 a 24:00 hrs) | | |
| **Persona encuestada:** | 1. Usuario ( ) 2. Acompañante ( ) 3. Ambos ( ) | | |

**<u>ESTUDIO SOBRE ACCIDENTES DE TRÁNSITO. C3T1 – Paciente</u>**

A. Tipo de lesión que sufrió en el momento del accidente *(Diagnóstico de Ingreso)*
*(Pregunta abierta)* *0) No sabe ( )*

1) ………………………………………………………………………………………………………………..
2) ………………………………………………………………………………………………………………..
3) ………………………………………………………………………………………………………………..
4) ………………………………………………………………………………………………………………..
5) ………………………………………………………………………………………………………………..

*(Escribir lo que menciona el paciente, y posteriormente contrastar o completar con lo escrito en la Historia Clínica)*

## I. CALIDAD Y RAPIDEZ DE ATENCIÓN LUEGO DE OCURRIDO EL ACCIDENTE:

**1. En el lugar del accidente, ¿Quién fue la primera persona en atenderlo?**

| | |
|---|---|
| 1 | Los bomberos *(Preguntar **1a**)* |
| 2 | Otra ambulancia pero no de los bomberos *(Preguntar **1a**)* |
| 3 | La policía o serenazgo *(Preguntar **1a**)* |
| 4 | Las personas cercanas al lugar del accidente *(Preguntar **1a**)* |
| 5 | Las personas que estaban conmigo en el momento del accidente *(Preguntar **1a**)* |
| 9 | No sabe/No responde/No recuerda (*NO LEER*). (*Pasar a pregunta **2***) |

**1a. La primera persona que lo atendió, ¿cuánto demoró aproximadamente en llegar al lugar del accidente desde ocurrido el mismo?:**

☐☐☐ mins NS/NR/No recuerda ( )

*(Si los primeros en atender fueron los bomberos o un servicio de ambulancia, pasar a pregunta **2**)*

**1b.**¿Y cuánto demoraron aproximadamente en llegar al lugar del accidente desde ocurrido el mismo, los bomberos u otro servicio de ambulancia?:

| | |
|---|---|
| 1 | Se demoraron ☐☐☐mins |
| 2 | Nunca llegaron |
| 9 | No sabe/No responde/No recuerda (*NO LEER*) |

**2. En general, con respecto a las personas que lo atendieron en el lugar del accidente y la atención que le brindaron.¿Usted se ha sentido….**

*(Marcar solo sobre las personas que lo atendieron)*

| | Muy Bien atendido | Bien Atendido | Mal Atendido | Muy Mal Atendido | NS/NR *(NO LEER)* |
|---|---|---|---|---|---|
| Bomberos | 1 | 2 | 3 | 4 | 9 |
| Otra Ambulancia | 1 | 2 | 3 | 4 | 9 |
| Policía | 1 | 2 | 3 | 4 | 9 |
| Otras personas | 1 | 2 | 3 | 4 | 9 |

## II. CALIDAD Y RAPIDEZ DE ATENCION EN EL HOSPITAL U OTRO ESTABLECIMIENTO DE SALUD

*(Si es necesario cambie "hospital" por "clínica" u otro)*

**3. Cuando los trasladaron del lugar del accidente hasta el establecimiento de salud donde lo atendieron. ¿Cuánto tiempo paso desde que ingreso al establecimiento de salud hasta que alguna persona (médico, enfermera u otra persona) lo llegara a atender?**

☐☐☐ mins NS/NR/No recuerda ( )

**4. ¿Quién fue la primera persona en atenderlo?**

| | |
|---|---|
| 1 | Un médico *(pasar a pregunta **5**)* |
| 2 | Una enfermera *(Preguntar **4a**)* |
| 3 | Otro persona que no era médico ni enfermera<br><br>*(especificar: ………………………………..…….)* *(Preguntar **4a**)* |
| 4 | No sabe/No responde/No se acuerda *(NO LEER) (pasar a pregunta **5**)* |

**4a. Si primero lo atendió una enfermera u otra persona. ¿Cuánto tiempo más demoró el médico en atenderlo?:**

| | |
|---|---|
| 1 | Se demoró ☐☐☐ mins |
| 2 | Nunca llegó |
| 9 | No sabe/No responde/No se acuerda *(NO LEER)* |

**5. Responda la alternativa que le parezca correcta con respecto a lo que usted <u>ESPERA ENCONTRAR DURANTE SU ATENCIÓN</u> en un hospital u otro establecimiento de salud**

*(Leer las alternativas de la Guía del Entrevistador y entregar Cartilla del Paciente)*

**Alternativas:** Del 1 al 7.

1 ….. 2 ….. 3 ….. 4 ….. 5 ….. 6 ….. 7
Muy en Desacuerdo Muy De Acuerdo

| | **Alternativas** | | | | | | |
|---|---|---|---|---|---|---|---|
| Alternativa 01E | 1 | 2 | 3 | 4 | 5 | 6 | 7 |
| Alternativa 02E | 1 | 2 | 3 | 4 | 5 | 6 | 7 |
| Alternativa 03E | 1 | 2 | 3 | 4 | 5 | 6 | 7 |
| Alternativa 04E | 1 | 2 | 3 | 4 | 5 | 6 | 7 |
| Alternativa 05E | 1 | 2 | 3 | 4 | 5 | 6 | 7 |
| Alternativa 06E | 1 | 2 | 3 | 4 | 5 | 6 | 7 |
| Alternativa 07E | 1 | 2 | 3 | 4 | 5 | 6 | 7 |
| Alternativa 08E | 1 | 2 | 3 | 4 | 5 | 6 | 7 |
| Alternativa 09E | 1 | 2 | 3 | 4 | 5 | 6 | 7 |
| Alternativa 10E | 1 | 2 | 3 | 4 | 5 | 6 | 7 |
| Alternativa 11E | 1 | 2 | 3 | 4 | 5 | 6 | 7 |
| Alternativa 12E | 1 | 2 | 3 | 4 | 5 | 6 | 7 |
| Alternativa 13E | 1 | 2 | 3 | 4 | 5 | 6 | 7 |
| Alternativa 14E | 1 | 2 | 3 | 4 | 5 | 6 | 7 |
| Alternativa 15E | 1 | 2 | 3 | 4 | 5 | 6 | 7 |
| Alternativa 16E | 1 | 2 | 3 | 4 | 5 | 6 | 7 |
| Alternativa 17E | 1 | 2 | 3 | 4 | 5 | 6 | 7 |
| Alternativa 18E | 1 | 2 | 3 | 4 | 5 | 6 | 7 |

6. **Responda la alternativa que le parezca correcta con respecto a lo que usted <u>PERCIBIÓ DURANTE SU ATENCIÓN</u> en el hospital u otro establecimiento de salud en donde fue atendido**

*(Leer las alternativas de la Guía del Entrevistador y entregar Cartilla del Paciente)*

**Alternativas:** Del 1 al 7.

1 ….. 2 ….. 3 ….. 4 ….. 5 ….. 6 ….. 7
Muy en Desacuerdo Muy De Acuerdo

| | Alternativas | | | | | | |
|---|---|---|---|---|---|---|---|
| Alternativa 01P | 1 | 2 | 3 | 4 | 5 | 6 | 7 |
| Alternativa 02P | 1 | 2 | 3 | 4 | 5 | 6 | 7 |
| Alternativa 03P | 1 | 2 | 3 | 4 | 5 | 6 | 7 |
| Alternativa 04P | 1 | 2 | 3 | 4 | 5 | 6 | 7 |
| Alternativa 05P | 1 | 2 | 3 | 4 | 5 | 6 | 7 |
| Alternativa 06P | 1 | 2 | 3 | 4 | 5 | 6 | 7 |
| Alternativa 07P | 1 | 2 | 3 | 4 | 5 | 6 | 7 |
| Alternativa 08P | 1 | 2 | 3 | 4 | 5 | 6 | 7 |
| Alternativa 09P | 1 | 2 | 3 | 4 | 5 | 6 | 7 |
| Alternativa 10P | 1 | 2 | 3 | 4 | 5 | 6 | 7 |
| Alternativa 11P | 1 | 2 | 3 | 4 | 5 | 6 | 7 |
| Alternativa 12P | 1 | 2 | 3 | 4 | 5 | 6 | 7 |
| Alternativa 13P | 1 | 2 | 3 | 4 | 5 | 6 | 7 |
| Alternativa 14P | 1 | 2 | 3 | 4 | 5 | 6 | 7 |
| Alternativa 15P | 1 | 2 | 3 | 4 | 5 | 6 | 7 |
| Alternativa 16P | 1 | 2 | 3 | 4 | 5 | 6 | 7 |
| Alternativa 17P | 1 | 2 | 3 | 4 | 5 | 6 | 7 |
| Alternativa 18P | 1 | 2 | 3 | 4 | 5 | 6 | 7 |

7. **En general, con respecto a la atención brindada en el hospital, ¿Usted se ha sentido….**

| | |
|---|---|
| 1 | Muy bien atendido |
| 2 | Bien atendido |
| 3 | Mal atendido |
| 4 | Muy mal atendido |
| 9 | No sabe/No responde/No se acuerda *(NO LEER)* |

8. **¿Le explicaron cuál era su diagnóstico o los problemas/enfermedades causadas por el accidente?**

| | |
|---|---|
| 1 | Sí *(Preguntar 8a)* |
| 2 | No *(pasar a pregunta 9)* |
| 9 | No sabe/No responde/No se acuerda (NO LEER) *(pasar a pregunta 9)* |

**8a. ¿Quién fue la persona que le explicó?:**

| | |
|---|---|
| 1 | Un médico |
| 2 | Una enfermera |
| 3 | Otro persona que no era médico ni enfermera |
| | *(especificar: ……………………..…….)* |
| 4 | No sabe/No responde/No se acuerda *(NO LEER)* |

9. **¿Y le explicaron qué es lo que tenían que hacer en ese momento o posteriormente para solucionar el diagnóstico o problema causado por el accidente de tránsito?**

| | |
|---|---|
| 1 | Sí |
| 2 | No |
| 9 | NS/NR (NO LEER) |

10. **¿Tiene usted algún conocimiento sobre lo que dice la ley con respecto a la obligación de los establecimientos de salud en dar atención a cualquier persona en caso de emergencias?**

| | |
|---|---|
| 1 | Sí *(Preguntar **10a**)* |
| 2 | No *(Pasar a pregunta **11**)* |
| 9 | No sabe/No responde/No se acuerda *(NO LEER) (Pasar a pregunta **11**)* |

**10a. Según lo que dice esa LEY, es decir:**

*(Leer las alternativas de la Guía del Entrevistador y entregar Cartilla del Paciente)*

¿Considera usted que lo atendieron en el establecimiento de salud bajo lo que dice dicha ley?

| | |
|---|---|
| 1 | Sí *(Pasar a pregunta **11**)* |
| 2 | No *(Preguntar **10b**)* |
| 9 | No sabe/No responde/No se acuerda *(NO LEER) (Pasar a pregunta **11**)* |

**10b. Si es NO, ¿En qué aspecto de la ley se equivocaron?**

*(Puede marcar varias alternativas)*

| | A | B | C | D |
|---|---|---|---|---|
| Se equivocaron en: | 1 | 2 | 3 | 4 |

11. **Cuando fue atendido en el establecimiento de salud luego del accidente de tránsito, ¿Con qué seguro fue atendido?**

| | |
|---|---|
| 1 | No tengo seguro o SOAT *(Preguntar **11a**)* |
| 2 | Con el SOAT, de mi vehículo o de otro *(Pasar a pregunta **12**)* |
| 3 | Con un seguro contra accidentes que no es SOAT *(Pasar a pregunta **12**)* |
| 4 | Con ambos, el SOAT y mi seguro contra accidentes *(Pasar a pregunta **12**)* |
| 5 | Contaba con seguro o SOAT pero no fui atendido con dicho seguro *(Preguntar **11b**)* |
| 9 | No sabe/No responde/No se acuerda *(NO* |

| | |
|---|---|
| | *LEER) (Pasar a pregunta **12**)* |

**11a.** Cómo Usted no contaba con seguro o SOAT, ¿quién pago la atención que le brindaron en ese establecimiento de salud?

*(Puede marcar varias alternativas)*

| | |
|---|---|
| 1 | Yo *(Pasar a pregunta **12**)* |
| 2 | Mis parientes o familiares *(Pasar a pregunta **12**)* |
| 3 | El chofer del vehículo involucrado en el accidente que no es el propietario *(Pasar a pregunta **12**)* |
| 4 | El chofer del vehículo involucrado en el accidente y además propietario *(Pasar a pregunta **12**)* |
| 5 | La persona (dueño) o empresa propietaria del vehículo involucrado en el accidente *(Pasar a pregunta **12**)* |
| 6 | Otra persona o institución *(Especificar: ………………………………………..)* *(Pasar a pregunta **12**)* |
| 9 | No sabe/No responde/No se acuerda (NO LEER) *(pasar a pregunta **12**)* |

**11b.** Si Ud. contaba con un seguro ¿Por qué no fue atendido con él?

| | |
|---|---|
| 1 | Mi seguro (o SOAT) no cubría mis lesiones *(Pasar a pregunta **12**)* |
| 2 | Mi seguro (o SOAT) no era válido en ese establecimiento de salud *(Pasar a pregunta **12**)* |
| 3 | Mi seguro (o SOAT) era válido, pero por problemas administrativos no pudieron atenderme *(Pasar a pregunta **12**)* |
| 4 | Mi seguro (o SOAT) se había vencido, no lo había pagado o no lo tenía *(Pasar a pregunta **12**)* |
| 5 | Otro *(Especificar ………………………………)* *(Pasar a pregunta **12**)* |
| 9 | No sabe/No responde/No se acuerda *(NO LEER)* *(Pasar a pregunta **12**)* |

**12. En su opinión ¿en general, qué es lo MÁS IMPORTANTE que debería mejorar el hospital donde fue atendido?**

| | |
|---|---|
| La rapidez de atención | 1 |
| El trato al paciente | 2 |
| Los conocimientos del médico | 3 |
| Los equipos y utensilios | 4 |
| La distribución de las instalaciones | 5 |
| Otro *(Especificar ……………………..………………)* | 6 |
| NS/NR | 9 |

**12a. Marque la opción que considere en SEGUNDO LUGAR DE IMPORTANCIA**

| | |
|---|---|
| La rapidez de atención | 1 |
| El trato al paciente | 2 |
| Los conocimientos del médico | 3 |
| Los equipos y utensilios | 4 |
| La distribución de las instalaciones | 5 |
| Otro *(Especificar …………………………………)* | 6 |
| NS/NR | 9 |

**¡Muchas gracias por su participación y colaboración!**

**Entrevista sobre atención pre e intra-hospitalaria de accidentes de tránsito. Paciente.**

**GUÍA DEL ENTREVISTADOR**

**PREGUNTA 5**

**Responda la alternativa que le parezca correcta con respecto a lo que usted ESPERA ENCONTRAR DURANTE SU ATENCIÓN en un hospital u otro establecimiento de salud**

**ALTERNATIVAS:** Del 1 al 7.

1 ….. 2 ….. 3 ….. 4 ….. 5 ….. 6 ….. 7
**Muy en Desacuerdo** **Muy De Acuerdo**

| | EXPECTATIVAS |
|---|---|
| Alternativa 01E | Un buen Hospital debe tener los instrumentos , equipos y aparatos necesarios |
| Alternativa 02E | El local de un buen Hospital debe ser agradable, estar limpio y ordenado |
| Alternativa 03E | Todos los que trabajan en un buen Hospital deben estar limpios y presentables |
| Alternativa 04E | Un buen Hospital debe ser fácil de ser identificado o reconocido, debe tener letreros, señales y lista de precios que orienten al paciente |
| Alternativa 05E | Un buen Hospital debe cumplir con lo que ofrece |
| Alternativa 06E | Un buen Hospital debe cumplir con el horario establecido |
| Alternativa 07E | Los trabajadores de un buen Hospital deben auxiliar y atender al paciente en el momento que lo necesita |
| Alternativa 08E | Un buen Hospital debe dar recibos según las tarifas y precios establecidos |
| Alternativa 09E | Un buen Hospital debe contar con los medicamentos y materiales |
| Alternativa 10E | En un buen Hospital, el personal debe informar a los pacientes a que hora aproximadamente van a ser atendidos |
| Alternativa 11E | Los trabajadores de un buen Hospital deben responder rápidamente a los pedidos y preocupaciones de los pacientes |
| Alternativa 12E | Los trabajadores de un buen hospital deben estar dispuestos a ayudar a los pacientes |
| Alternativa 13E | Los pacientes deben sentir que pueden confiar en los trabajadores de un buen hospital |
| Alternativa 14E | Los trabajadores de un buen Hospital deben conocer lo necesario para informar y orientar a los pacientes |
| Alternativa 15E | Los trabajadores de un buen Hospital deben ser amables y atentos |
| Alternativa 16E | Los trabajadores de un buen Hospital deben apoyarse entre compañeros para dar la mejor atención posible |
| Alternativa 17E | Los trabajadores de un buen hospital deben ser capaces de atender a cada paciente según su situación y características particulares |
| Alternativa 18E | En un buen hospital se debe pensar primero en el paciente |

**PREGUNTA 6**

**Responda la alternativa que le parezca correcta con respecto a lo que usted PERCIBIÓ DURANTE SU ATENCIÓN en el hospital u otro establecimiento de salud en donde fue atendido**

**ALTERNATIVAS:** Del 1 al 7.

1 ….. 2 ….. 3 ….. 4 ….. 5 ….. 6 ….. 7
**Muy en Desacuerdo** **Muy De Acuerdo**

| | PERCEPCIONES |
|---|---|
| Alternativa 01P | Este Hospital tiene los instrumentos, equipos y aparatos necesarios |
| Alternativa 02P | Este Hospital es agradable, limpio y ordenado |
| Alternativa 03P | Todos los que trabajan en este Hospital están limpios y presentables |
| Alternativa 04P | Este Hospital es fácil de ser identificado o reconocido, tiene letreros, señales y lista de precios que orienten al paciente |
| Alternativa 05P | Este Hospital cumple con lo que ofrece |
| Alternativa 06P | Este Hospital cumple con el horario establecido |
| Alternativa 07P | Los trabajadores de este Hospital auxilian y atienden al paciente en el momento que lo necesita |
| Alternativa 08P | En este Hospital se da recibos según las tarifas y precios establecidos |
| Alternativa 09P | Este Hospital cuenta con los medicamentos y materiales necesarios |
| Alternativa 10P | El personal de este Hospital informa a los pacientes y a sus acompañantes a que hora aproximadamente van a ser atendidos |
| Alternativa 11P | Los trabajadores de este Hospital responden rápidamente a los pedidos y preocupaciones de los pacientes |
| Alternativa 12P | Los trabajadores de este Hospital están dispuestos a ayudar a los pacientes |
| Alternativa 13P | Los pacientes sienten confianza en los trabajadores de este Hospital |
| Alternativa 14P | Los trabajadores de este Hospital saben lo necesario para informar y orientar a los pacientes |
| Alternativa 15P | Los trabajadores de este Hospital son amables y atentos |
| Alternativa 16P | Los trabajadores de este Hospital se ayudan entre compañeros para dar la mejor atención posible |
| Alternativa 17P | Los trabajadores de un buen hospital deben ser capaces de atender a cada paciente según su situación y características particulares |
| Alternativa 18P | En este Hospital se piensa primero en el paciente |

**PREGUNTA 10A**

**11a. Según lo que dice esa LEY, es decir:**

**A.** Todos los establecimientos de salud, sin excepción, están obligados a prestar atención inmediata a toda persona en situación de emergencia.
**B.** La determinación de la condición de emergencia médica es realizada por el profesional médico encargado de la atención
**C.** Cuando el establecimiento de salud no pueda brindar los recursos necesarios para la atención especializada que el paciente requiera, se procederá a llamar a un profesional especialista o se transferirá al paciente a otro establecimiento
**D.** El pago por concepto de atención de la emergencia, se realizará posterior a la atención

**C3 T1**
**Entrevista sobre atención pre e intra-hospitalaria de accidentes de tránsito. Paciente.**

**FICHA PARA EL PACIENTE**

**PREGUNTA 5**

**Responda la alternativa que le parezca correcta con respecto a lo que usted ESPERA ENCONTRAR DURANTE SU ATENCIÓN en un hospital u otro establecimiento de salud**

**ALTERNATIVAS:** Del 1 al 7.

1 ….. 2 ….. 3 ….. 4 ….. 5 ….. 6 ….. 7
**Muy en Desacuerdo** **Muy De Acuerdo**

**PREGUNTA 6**

**Responda la alternativa que le parezca correcta con respecto a lo que usted PERCIBIÓ DURANTE SU ATENCIÓN en el hospital u otro establecimiento de salud en donde fue atendido**

**ALTERNATIVAS:** Del 1 al 7.

1 ….. 2 ….. 3 ….. 4 ….. 5 ….. 6 ….. 7
**Muy en Desacuerdo** **Muy De Acuerdo**

**PREGUNTA 10A y 10B**

**Según lo que dice esa LEY, es decir:**

- **A.** Todos los establecimientos de salud, sin excepción, están obligados a prestar atención inmediata a toda persona en situación de emergencia.
- **B.** La determinación de la condición de emergencia médica es realizada por el profesional médico encargado de la atención
- **C.** Cuando el establecimiento de salud no pueda brindar los recursos necesarios para la atención especializada que el paciente requiera, se procederá a llamar a un profesional especialista o se transferirá al paciente a otro establecimiento
- **D.** El pago por concepto de atención de la emergencia, se realizará posterior a la atención
