# Supplementary material for: Patient perspectives on the promptness and quality of care of road traffic incident victims in Peru: a cross-sectional, active surveillance study
Source: F1000Res. 2013 Aug 9;2:167. [Version 1] doi: 10.12688/f1000research.2-167.v1 (PMC3814912; doi:10.12688/f1000research.2-167.v1)
Supplement: Peer review history

# Peer review: Referee response for version 1

**Published:** 2013-09-05

**Authors:** Naoto Morimura

**Affiliations:** 1 Department of Emergency Medicine, Yokohama City University School of Medicine, Yokohama, Japan

This report is very important for assessing and recognizing the present status of trauma systems in the region. 

I believe an analysis of mortality however, for example an investigation of preventable trauma death by trauma scoring, will give this article greater support. In addition to the evaluation of access time to the hospital and patient’s satisfaction of this study, further study based on the outcome can enable the quality of the trauma management.

I have read this submission. I believe that I have an appropriate level of expertise to confirm that it is of an acceptable scientific standard.

# Peer review: Referee response for version 1

**Published:** 2013-08-30

**Authors:** Andrej Michalsen

**Affiliations:** 1 Department of Anaesthesiology and Critical Care Medicine, Tettnang Hospital, Tettnang, Germany

The article describes the promptness and quality of care as perceived by approximately 640 road traffic incident victims at 8 healthcare facilities in Peru within 4 weeks. Both the promptness and the quality leave room for improvement because the average response time is far longer than internationally recommended and the quality of in-hospital services is considered low by those patients who took part in the surveillance. The abstract provides a reasonable summary of the article, except perhaps where promptness is rated as ' *adequate overall*'; a better description here might be "adequate under the given circumstances".

The strength of the research is the clear methodology, which is comprehensively explained and followed. Without reliable data there will not be a sound basis for policy makers to decide on the allocation of scarce resources. Perhaps, the study period could have been longer than just four weeks to increase the validity of the results. Also, the goals for improving promptness of care lean especially towards U.S. standards without explaining their (alleged) superiority over standards in other parts of the world. 

The conclusions of the study appear balanced and justified. A replication of the study at more sites in Peru and beyond would be helpful to make policy makers aware of the importance of improvements in healthcare for injury victims as injury is one of the leading causes of morbidity and mortality worldwide.

I have read this submission. I believe that I have an appropriate level of expertise to confirm that it is of an acceptable scientific standard.
